# *Rhinogobius mengyangensis*: a new species of freshwater goby (Teleostei, Gobiidae) from Sichuan Province, southwestern China

**DOI:** 10.3897/zookeys.1280.188225

**Published:** 2026-05-28

**Authors:** Xin Liu, Zhong-Guang Chen, Yi-Fan Shu, Jun-Hao Huang, Kai Liu, Yi Yu

**Affiliations:** 1 The Fishery Institute of the Sichuan Academy of Agricultural Sciences, Chengdu, Sichuan 611730, China The Museum of Aquatic Organisms, Institute of Hydrobiology, Chinese Academy of Sciences Wuhan China; 2 Department of Biochemistry, McGill University, Montréal, Québec H3T 1E2, Canada Department of Biochemistry, McGill University Montréal Canada; 3 School of Life Sciences, Nanchang University, Nanchang, Jiangxi 330031, China College of Animal and Technology, Sichuan Agricultural University Chengdu China; 4 School of Nursing and Health, Zhengzhou University, Zhengzhou, Henan 450001, China School of Life Sciences, Nanchang University Nanchang China; 5 The Museum of Aquatic Organisms, Institute of Hydrobiology, Chinese Academy of Sciences, Wuhan, Hubei 430072, China School of Nursing and Health, Zhengzhou University Zhengzhou China; 6 College of Animal and Technology, Sichuan Agricultural University, Chengdu, Sichuan 611130, China The Fishery Institute of the Sichuan Academy of Agricultural Sciences Chengdu China

**Keywords:** Biodiversity, freshwater, phylogeny, taxonomy

## Abstract

A new species of freshwater goby, *Rhinogobius mengyangensis***sp. nov**., is described from the upper Changjiang River basin in Sichuan Province, China, based on comparative morphology and molecular phylogeny. The new species is sister to *Rhinogobius szechuanensis* Tchang, 1939 and can be distinguished from its congeners by the following combination of characters: absence of sensory canals and pores on head; cheeks and operculum without spots or stripes; second dorsal fin with 9–11 longitudinal rows of inverted V-shaped or dash-like brown markings; incomplete brownish edges on flank scales below second dorsal fin; branchiostegal membrane bright yellow.

## Introduction

The goby genus *Rhinogobius* Gill, 1859 is mainly distributed in freshwater in East Asia and Southeast Asia (Akihito et al. 2000). In mainland China, more than 50 valid species of this genus have been recorded (Wanghe et al. 2020). It is the most diverse genus of freshwater goby in the region, and new species are being constantly described (Wanghe et al. 2020; Chen et al. 2022a; Chen et al. 2022b; Wang and Chen 2022; Li et al. 2024; Zhou et al. 2024). *Rhinogobius szechuanensis* Tchang, 1939 is a small-bodied benthic goby, which represents the non-diadromous ecotype according to its life histories. It inhabits mountain streams, plain creeks, and main streams of the Minjiang River basin (Wu and Zhong 2008; Liu et al. 2023), suggesting an extensive adaptability. Compared with congeneric species, this species is uniquely characterized by an absence of sensory canals and sensory pores, an absence of spots or stripes on cheek and snout, and flank scales each with complete brownish edges forming a reticulate pattern (Wu and Zhong 2008). Due to its striking body colouration, *R. szechuanensis* is popularly kept as a pet fish. Commercial overfishing has resulted in a significant decline in some populations. This species exhibits two colour types, one red and the other yellow, which have long been considered conspecific (Guo et al. 2021).

In the present study, we conducted a detailed survey of *Rhinogobius* species in the Minjiang and Tuojiang River basins of Sichuan, China, in 2023–2024. Morphological comparison and molecular phylogeny reveal the existence of a yet-to-be-described species closely related to *R. szechuanensis* (red type). Here we describe it as a new species.

## Materials and methods

Specimens were collected by hand-net and fish trap. Fifteen specimens were fixed in 8% formalin for morphological study, and six specimens were fixed in 99% ethanol for molecular phylogeny. Morphometric and meristic data were obtained and analysed following the methods of Chen and Fang (2006) and Miller (1988), respectively. Photographs were taken by Sony A7C II and edited in Adobe Photoshop CC 2015 (Adobe, San Jose, USA). Maps were made in ArcGIS Pro (Esri, Redlands, USA).

Total DNA was purified from caudal fin with TIANamp Genomic DNA Kit (Tiangen, China). To reduce mutations during polymerase chain reactions (PCR), we used Phanta Max Master Mix (which contained Super-Fidelity DNA Polymerase; Vazyme, China) for *Cyt b* gene amplification. PCR systems, conditions and primer pairs were followed Wanghe et al. (Wanghe et al. 2020). The sequences used in this study were shown in Table 1. Phylogenies were reconstructed by the *Cyt b* dataset using maximum likelihood (ML) and Bayesian inference (BI). *Tridentiger kuroiwae* Jordan & Tanaka, 1927 was used as the outgroup for rooting the trees. The best-fit model for gene and gene partition was calculated by PartitionFinder2 v. 1.1 (Lanfear et al. 2017), based on the corrected Akaike Information Criterion (AICc) and using a heuristic search algorithm. The program proposed the division of the concatenated dataset into one partition, and the best-fit model was determined to be GTR+I+G. ML analyses were performed in IQ-TREE v. 1.6.12 (Minh et al. 2013) using the Ultrafast bootstrap approach (Minh et al. 2013) with 10,000 iterations. Bayesian inference (BI) analysis was conducted in MrBayes v. 3.2.6 (Ronquist et al. 2012). Four simultaneous runs with four independent Markov Chain Monte Carlo (MCMC) were implemented for 10 million generations, and trees were sampled every 10,000 generations with a burn-in of 25%. The convergence was checked with the average standard deviation of split frequencies < 0.01 and the potential scale reduction factor (PSRF) ~1. Trees were visualized in FigTree v. 1.4.3 (http://tree.bio.ed.ac.uk/software/figtree/).

**Table 1.** GenBank accession numbers of sequences used in this study.

Species	Accession numbers	Locality	References
* R. mengyangensis* sp. nov. MY	PZ316365	Mengyang Town, Chengdu, China	This study
* R. mengyangensis* sp. nov. MS	PZ316366	Mingshan District, Yaan, China	This study
* R. mengyangensis* sp. nov. JJ1	PZ316367	Jiajiang County, Leshan, China	This study
* R. mengyangensis* sp. nov. JJ1	PZ316364	Jiajiang County, Leshan, China	This study
* R. mengyangensis* sp. nov. PZ1	PZ316368	Pengzhou City, Chengdu, China	This study
* R. mengyangensis* sp. nov. PZ2	PZ316369	Pengzhou City, Chengdu, China	This study
* R. mengyangensis* sp. nov. DY	PZ316363	Dayi County, Chengdu, China	This study
* R. szechuanensis* CZ	PZ316356	Chongzhou City, Chengdu, China	This study
* R. szechuanensis* YC	PZ316360	Yucheng District, Yaan, China	This study
* R. szechuanensis* DY	PZ316357	Dayi County, Chengdu, China	This study
* R. szechuanensis* QL	PZ316362	Qionglai City, Chengdu, China	This study
* R. szechuanensis* DJY	NC062786	Dujiangyang City, Chengdu, China	Liu et al. 2023
* R. szechuanensis* WJ	PZ316359	Wenjiang District, Chengdu, China	This study
* R. szechuanensis* EM	PZ316358	Emeishan City, Leshan, China	This study
* R. houheensis*	MK353341		Wanghe et al. 2020
* R. davidi*	OM617724		Song et al. 2023b
* R. muzunoi*	AB988936		Yamasaki et al. 2015
* R. brunneus*	NC028435		GenBank
* R. fluviatilis*	AB988917		Yamasaki et al. 2015
* R. kurodai*	AB988981		Yamasaki et al. 2015
* R. nagoyae*	AB988955		Yamasaki et al. 2015
* R. * sp.	AY645716		Cheng et al. 2005
* R. candidianus*	AY137601		GenBank
* R. formosanus*	MN549279		GenBank
* R. ogasawaraensis*	AB988950		Yamasaki et al. 2015
* R. biwaensis*	AB988982		Yamasaki et al. 2015
* R. rubromaculatus*	AY137603		GenBank
* R. maculagenys*	OK545540		Hu et al. 2023
* R. niger*	OM791349		GenBank
* R. lentiginis*	OM617725		Song et al. 2023a
* R. leavelli*	AB988976		Yamasaki et al. 2015
* R. cliffordpopei*	MK204741		GenBank
* R. virgigena*	AB988977		Yamasaki et al. 2015
* R. duospilus*	MH127918		Tan et al. 2020
* R. changtinensis*	KF929300		Huang et al. 2013
* R. filamentosus*	OM678440		Chen et al. 2022a
* R. estrellae*	LC648291		Maeda et al. 2021
* R. tandikan*	LC648297		Maeda et al. 2021
* R. similis*	AB988913		Yamasaki et al. 2015
* Tridentiger kuroiwae*	LC653489		Maeda et al. 2021

Abbreviations. **IHB**: Museum of Aquatic Organisms, Institute of Hydrobiology, Chinese Academy of Sciences (Wuhan, Hubei, China); **SAFS**: Museum of Aquatic Organisms, Sichuan Academy of Forestry Sciences (Chengdu, Sichuan, China); **SCSCS**: the Fishery Institute of the Sichuan Academy of Agricultural Sciences.

## Results

### Phylogenetic analyses

**Figure 1.**
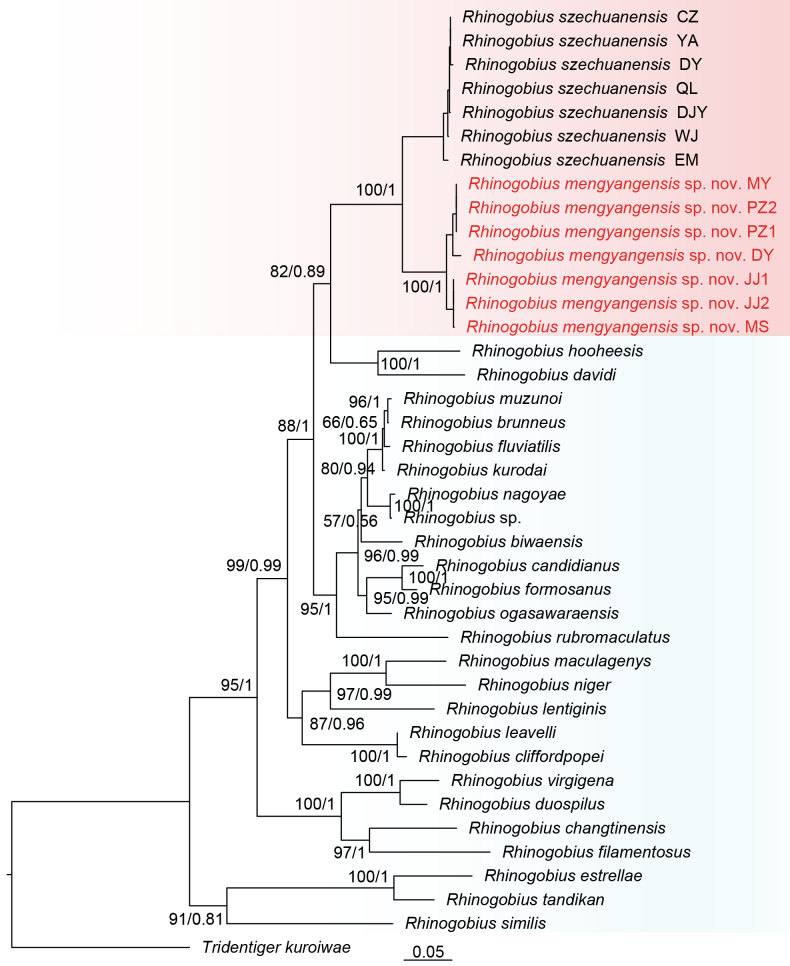
Maximum-likelihood tree and Bayesian inference tree of *Rhinogobius mengyangensis* sp. nov. inferred from *Cyt b* gene sequences. Bootstrap supports/posterior probabilities are shown on the left/right of nodes on the tree. The locality of *Rhinogobius mengyangensis* sp. nov. and *Rhinogobius szechuanensis* are list in Table 1.

The sequence dataset consisting of 39 *Cyt b* sequences from 28 species, including one outgroup taxon (*Tridentiger kuroiwae* Jordan & Tanaka, 1927), was employed for phylogenetic analyses (Table 1). The alignment of the *Cyt b* genes had a length of 1141 characters. Within these alignments, 467 sites were variable, and 387 sites were parsimony informative. The Bayesian and maximum-likelihood analyses produced largely similar phylogenies (Fig. 1). The new species is sister to *R. szechuanensis* with strong support (BS/PP = 100/1.00). The genetic distance *between R. mengyangensis* sp. nov. and *R. szechuanensis* in the *Cyt b* gene was 0.07.

### Systematics

#### Rhinogobius mengyangensis sp. nov.

Taxon classificationAnimaliaPerciformesGobiidae

E81ECBBC-BBB8-5648-B8A4-BC629D466CF0

https://zoobank.org/E41FC719-3FEE-403F-BDCE-BACDF51251A9

**Figure 2.**
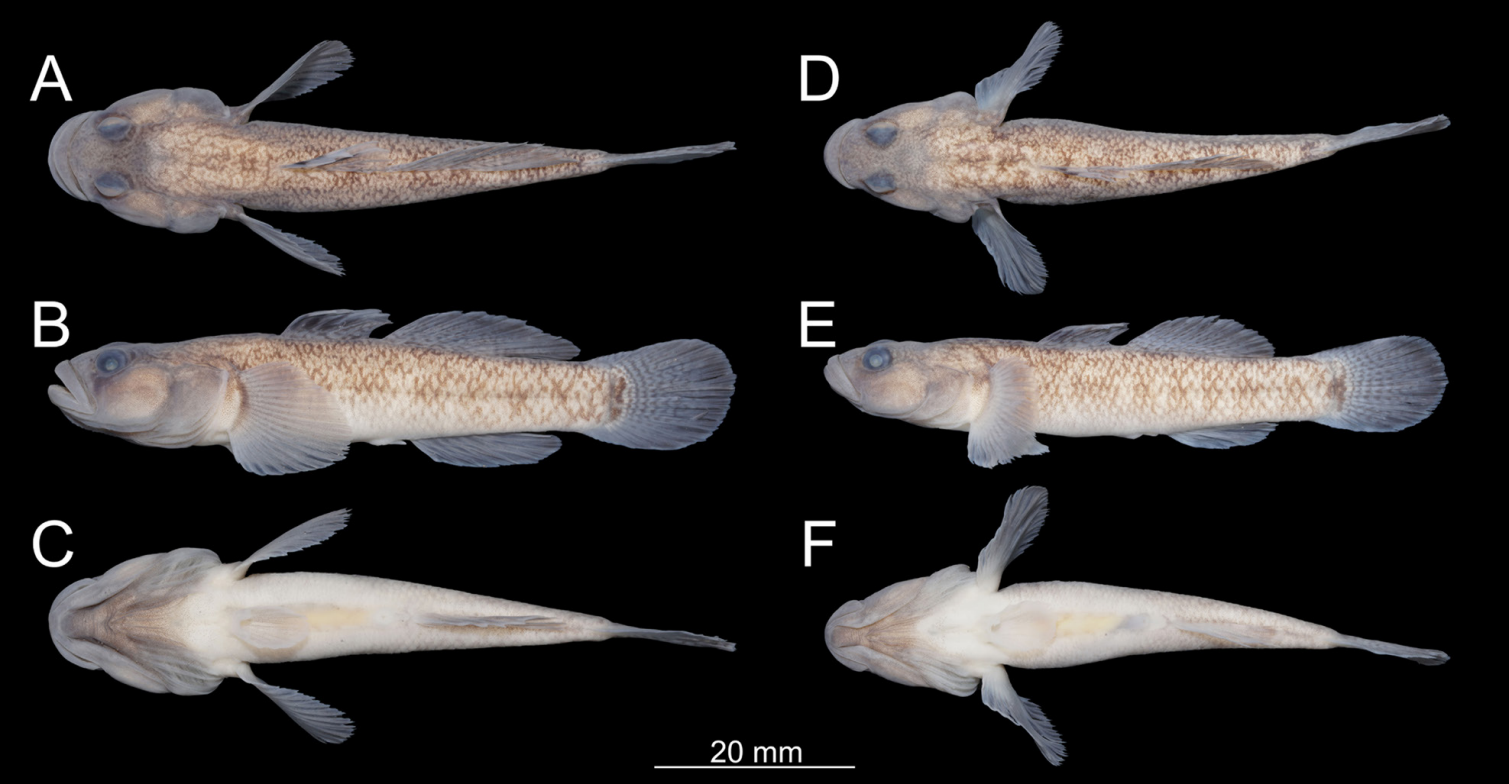
*
Rhinogobius mengyangensis* sp. nov. **A–C**. Holotype, IHB 0202506006; **D–F**. Paratype, IHB 0202506007.

**Figure 3.**
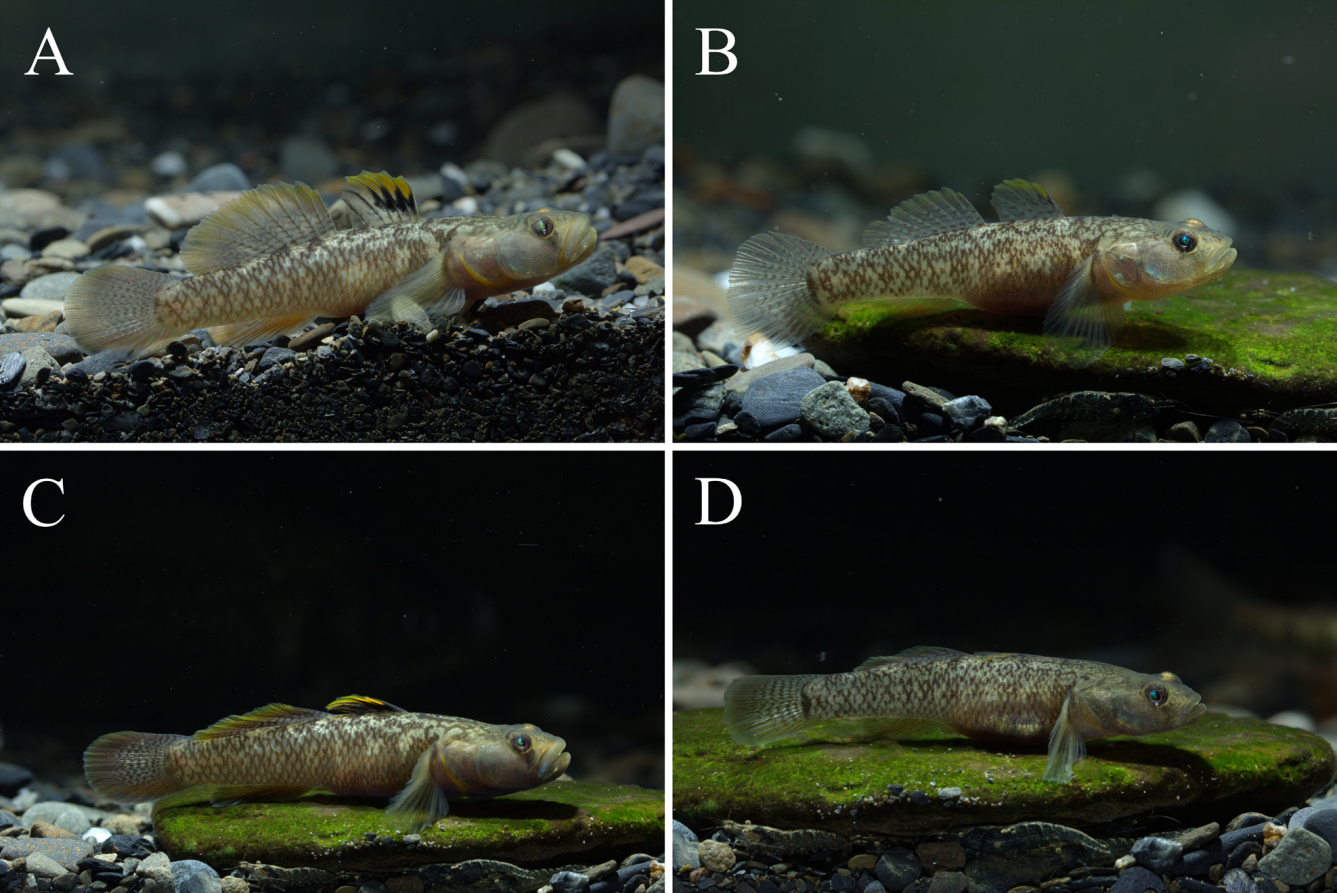
*
Rhinogobius mengyangensis* sp. nov. alive. **A**. Male, Tuojiang River basin (holotype, IHB 0202506006); **B**. Female, Tuojiang River basin (Paratype, IHB 0202506007); **C**. Male, Qingyijiang River basin; **D**. Female, Qingyijiang River basin.

**Figure 4.**
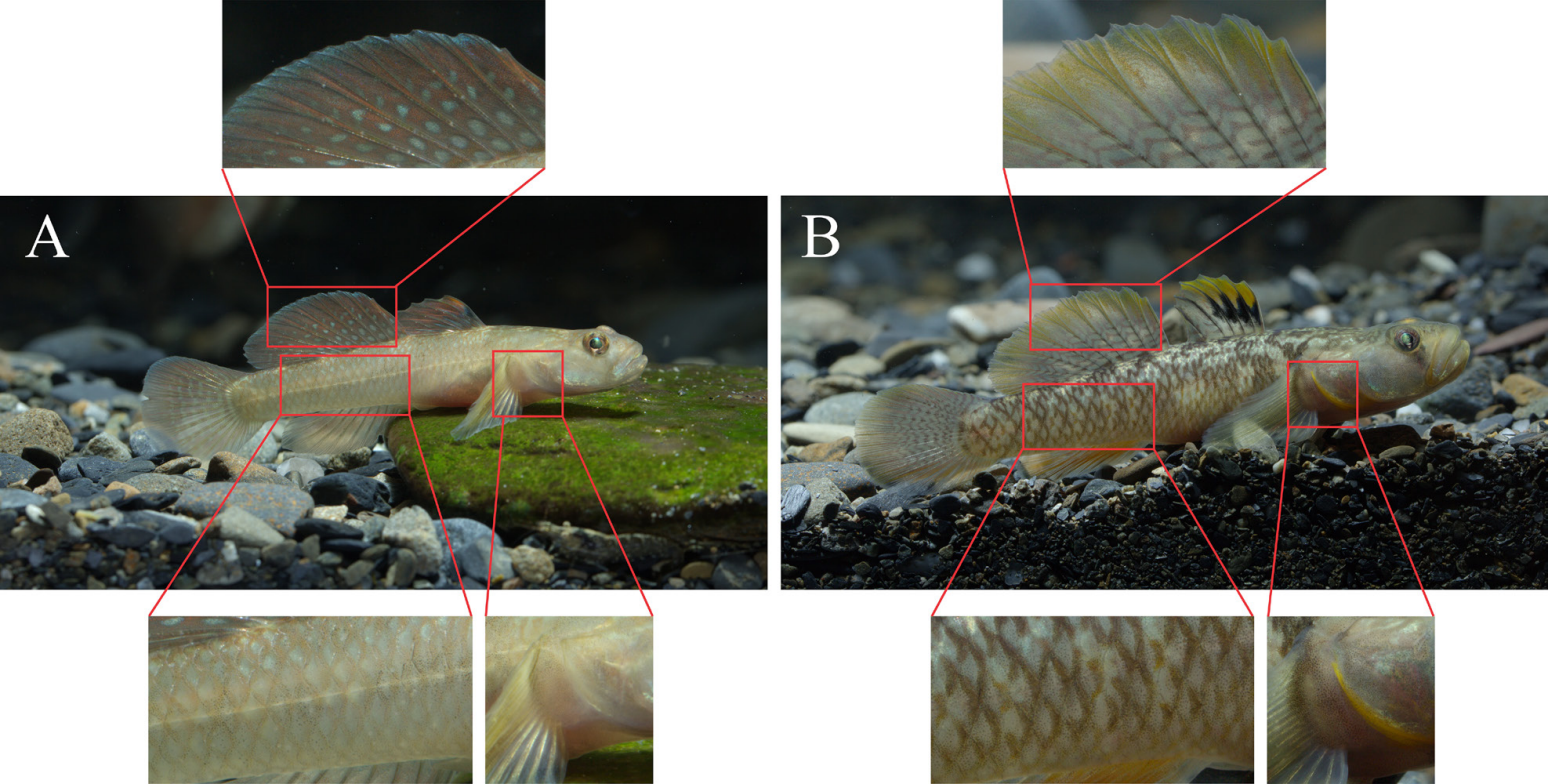
Comparison of the coloration between *Rhinogobius szechuanensis* and *Rhinogobius mengyangensis* sp. nov. **A**. *Rhinogobius szechuanensis*; **B**. *Rhinogobius mengyangensis* sp. nov.

**Figure 5.**
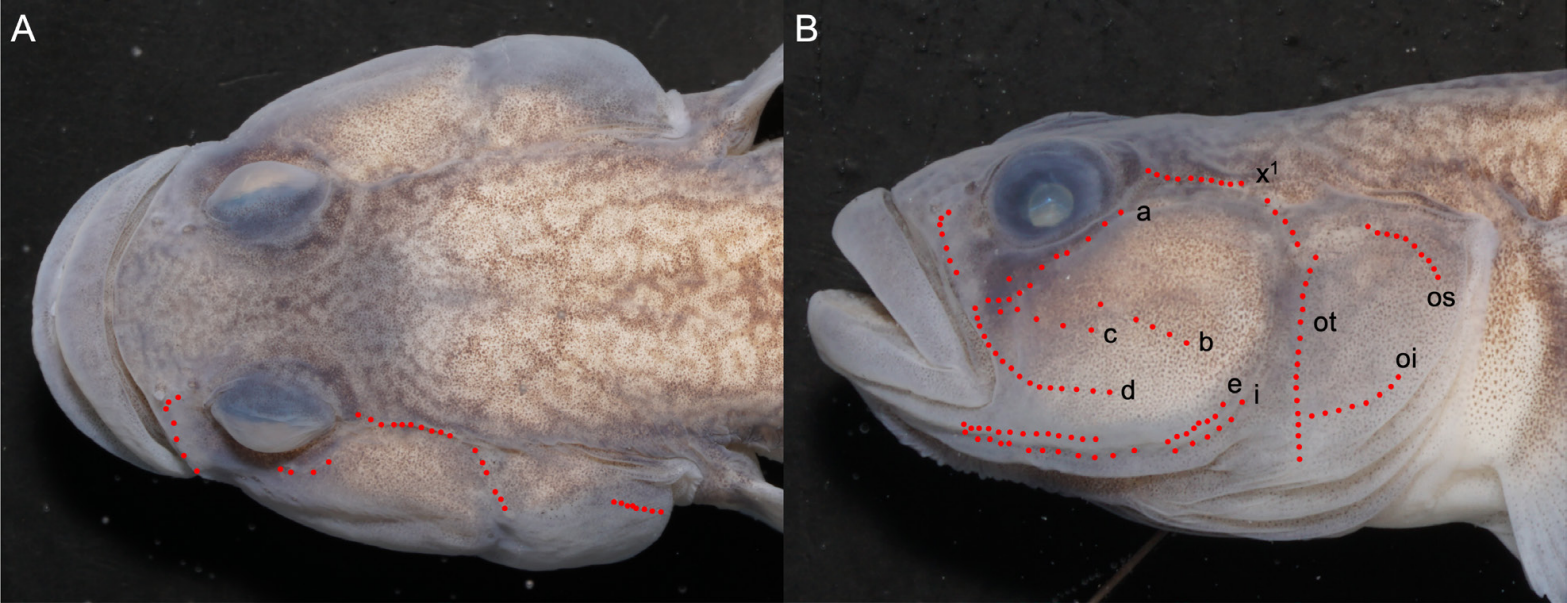
Sensory papillae of *Rhinogobius mengyangensis* sp. nov. (holotype, IHB 0202506006). **A**. Dorsal view; **B**. Lateral view.

**Figure 6.**
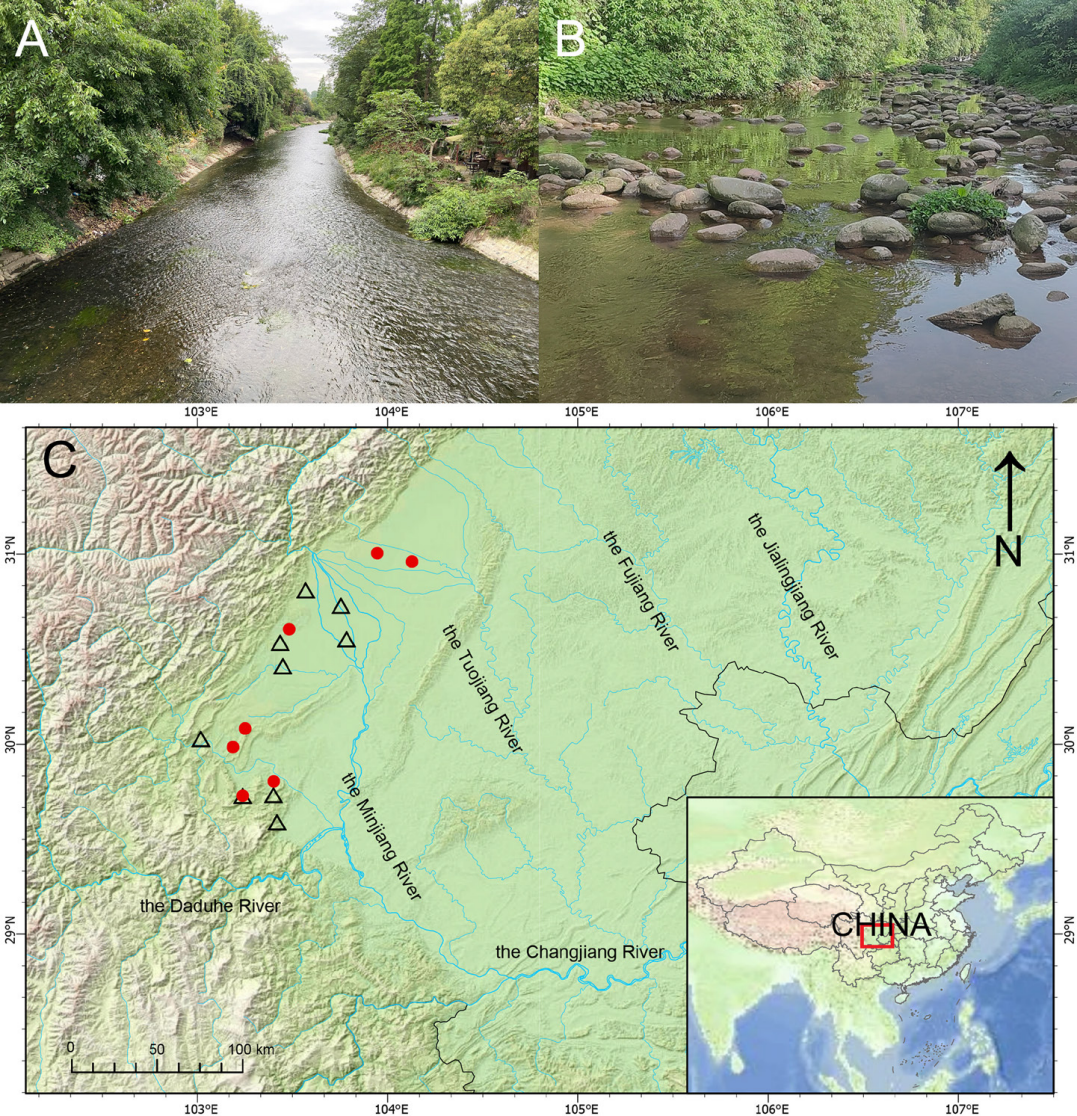
Distribution and habitat of *Rhinogobius mengyangensis* sp. nov. and *Rhinogobius szechuanensis*. **A**. The Mengyang River at Hengyang Town, Tuojiang River basin; **B**. The Mingshanhe River at Mingshan District, Qingyijiang River basin; **C**. Circles represent the distribution sites of *Rhinogobius mengyangensis*, while triangles represent the distribution sites of *Rhinogobius szechuanensis*.

Figs 1, 2, 3, 4, 5, 6; Tables 1, 2

##### Holotype.

• IHB 0202506006, male, 53.1 mm (Fig. 2); Mengyang River [濛阳河], Mengyang Town [濛阳镇], Pengzhou City [彭州市], Chengdu City [成都市], Sichuan Province, China, 30°57'37"N, 104°07'39"E, leg. Shuai Zhang, November 03, 2023.

##### Paratypes (*n* = 14).

• IHB 0202506007, female, 49.5 mm (Fig. 2), other information same as holotype; • IHB 0202506008–09, male, 38.66–54.92 mm, Mingshanhe River [名山河], Mingshan District [名山区], Yaan City [雅安市], Sichuan Province, China, 30°04'12"N, 103°06'34"E, leg. Shuai Zhang, August 25, 2023; • IHB 0202506010–12, female, 37.94–40.10 mm, Mingshanhe River [名山河], Mingshan District [名山区], Yaan City [雅安市], Sichuan Province, China, 30°04'12"N, 103°06'34"E, leg. Shuai Zhang, August 25, 2023; • SCSCS20250601–03, male, 46.90–59.32 mm, Baituhe River [百土河], Pengzhou City [彭州市], Chengdu City [成都市], Sichuan Province, China, 30°57'21"N, 104°05'26"E, leg. Shuai Zhang, July 30, 2023; • SCSCS20250604–05, male, 43.80–46.70 mm, Aihe River [矮河], Dayi County [大邑县], Chengdu City [成都市], Sichuan Province, China, 30°38'41"N, 103°33'52"E, leg. Shuai Zhang, August 12, 2024; • SCSCS20250606–07, female, 47.4–51.2 mm, Aihe River [矮河], Dayi County [大邑县], Chengdu City [成都市], Sichuan Province, China, 30°38'41"N, 103°33'52"E, leg. Shuai Zhang, August 12, 2024; • SCSCS20250608, male, 43.40 mm, Chuanxihe River [川溪河], Huatou County [华头镇], Jiajiang County [夹江县], Leshan City [乐山市], Sichuan Province, China, 29°44'02"N, 103°23'58"E, leg. Shuai Zhang and Wang Song, August 12, 2024.

##### Non-types.

• SAFS 230001, Mengyang River [濛阳河], Mengyang Town [濛阳镇], Pengzhou City [彭州市], Chengdu City [成都市], Sichuan Province, China, 30°57'37"N, 104°07'39"E, leg. Shuai Zhang, November 3, 2023; • SAFS 230002–03, Baituhe River [百土河], Pengzhou City [彭州市], Chengdu City [成都市], Sichuan Province, China, 30°57'21"N, 104°05'26"E, leg. Shuai Zhang, July 30, 2023; • SAFS 230004–05, Chuanxihe River [川溪河], Huatou Twon [华头镇], Jiajiang County [夹江县], Leshan City [乐山市], Sichuan Province, China, 29°44'02"N, 103°23'58"E leg. Shuai Zhang and Wang Song, August 12, 2024; • SAFS 230006, Mingshanhe River [名山河], Mingshan District [名山区], Yaan City [雅安市], Sichuan Province, China, 30°04'12"N, ​103°06'34"E, leg. Shuai Zhang, August 25, 2023; • SAFS 230007, Aihe River [矮河], Dayi County [大邑县], Chengdu City [成都市], Sichuan Province, China, 30°38'41"​​N, 103°33'52"E, leg. Shuai Zhang, August 12, 2024.

##### Diagnosis.

*Rhinogobius mengyangensis* sp. nov. can be distinguished from all congeners except *R. szechuanensis* by the following combination of features: absence of sensory canals and sensory pores on head; cheeks and operculum without spots or stripes (Fig. 3). It can be distinguished from *R. szechuanensis* in having the second dorsal fin with 9–11 longitudinal rows of inverted V-shaped or dash-like brown markings (vs with blue spots, some coalescing into vertical bars), incomplete brownish edges on flank scales below second dorsal fin (vs with complete edges), and a bright-yellow branchiostegal membrane (vs white or blue) (Fig. 4).

##### Description.

Morphometric measurements and meristic counts are provided in Table 2. see Figs 2, 3, and 5 for lateral body.

**Table 2.** Morphological and meristic measurements of *Rhinogobius mengyangensis* sp. nov.

Morphometrics characters	Holotype	Min–max (mean)	
Sex	Male	Male (*n* = 9)	Female (*n* = 6)
Standard length (mm)	53.17	38.66–59.32 (48.44)	37.94–51.20 (43.98)
Head length (mm)	16.87	11.50–18.58 (15.02)	10.50–16.52 (13.23)
Percent standard length (%)			
Head length	31.73	28.92–33.83 (30.92)	26.18–32.58 (30.01)
Predorsal length	40.42	38.80–43.18 (40.28)	38.15–42.61 (39.74)
Snout to second dorsal fin origin	58.89	55.82–60.42 (58.65)	57.61–63.65 (59.82)
Snout to anus	63.55	55.82–64.05 (60.75)	60.55–66.16 (62.71)
Prepelvic length	30.30	29.95–34.41 (31.15)	25.69–33.36 (30.42)
Caudal peduncle length	19.86	18.95–24.21 (21.53)	19.95–24.88 (21.81)
Caudal peduncle depth	13.11	10.92–13.11 (12.19)	11.60–13.05 (12.21)
First dorsal fin base	18.79	14.89–18.79 (16.47)	14.34–16.20 (15.36)
Second dorsal fin base	18.83	17.58–25.87 (20.61)	19.71–23.30 (20.38)
Anal fin base	14.90	10.28–17.04 (13.47)	12.68–18.70 (16.46)
Caudal fin length	21.12	20.55–24.85 (21.78)	20.19–23.63 (21.88)
Pectoral fin length	22.23	20.38–23.53 (21.97)	20.45–23.25 (22.18)
Pelvic fin length	14.99	11.00–16.50 (14.81)	12.92–16.52 (15.87)
Body depth of pelvic fin origin	16.02	14.49–19.52 (17.02)	13.34–21.81 (16.57)
Body depth of anal fin origin	15.82	14.43–18.76 (16.22)	13.34–19.51 (15.98)
Pelvic fin origin to anus	32.20	25.87–32.23 (28.29)	28.05–32.92 (31.53)
Head depth	18.11	14.02–18.71 (16.60)	12.91–17.93 (14.77)
Percent head length (%)			
Snout length	25.31	19.01–25.31 (22.73)	19.42–26.67 (24.28)
Eye diameter	19.62	15.33–20.71 (18.77)	17.11–23.81 (21.69)
Cheek depth	26.32	16.90–31.88 (22.82)	16.67–25.93 (19.67)
Postorbital length	60.58	53.52–60.70 (56.62)	54.00–60.95 (57.79)
Lower jaw length	38.65	22.78–39.78 (30.93)	25.54–38.38 (28.68)
Interorbital width	20.98	9.57–22.28 (17.28)	8.25–22.48 (15.36)
Head width in maximum	78.30	63.38–78.91 (70.39)	57.93–71.43 (64.64)
Meristic counts			
First dorsal fins	VI	VI (15)	
Second dorsal fins	I, 9	I, 8 (2), I, 9 (11), I, 10 (2)	
Anal fins	I, 8	I, 7 (10), I, 8 (3), I, 9 (2)	
Pectoral fins	19	16 (5), 17 (2), 19, (1)20 (5), 21 (2)	
Longitudinal scales	31	30 (1), 31 (2), 32 (4), 33 (4), 34 (2), 35 (2)	
Transverse scales	10	9 (11), 10 (4)	
Predorsal scales	6	5 (2), 6 (2), 8 (2)	
Vertebrae	27		

***Fins and Vertebrae***. First dorsal fin VI; 3rd and 4th rays longest, serrate during male breeding period. Second dorsal fin rays I/8–I/10 (mostly I/9). Anal fin rays I/7–I/8 (Table 2); origin below 2nd–3rd branched ray of second dorsal fin (Table 2). Pectoral fin rays 16–21 (Table 2). Pelvic fin rays I/5, elliptical. Total vertebrae 27. ***Scales***. Body covered with moderately large ctenoid scales; anterior trunk scales reduced. Almost whole head and pelvic base region naked. Longitudinal scale series counted 30–35 (Table 2). Transverse scale series 9–10 (Table 2). ***Sensory papillae***. Sensory papillae illustrated in Figs 5A and B. Row a obliquely uniserial, beneath orbit. Row d elongate, reaching vertical through posterior orbital margin. Rows a, c, and d converging at eye–upper jaw junction. Rows e and i with paired papillae. Row x1 postorbital supracheek. Opercular papillae ot, oi, and os present. Sensory papillae well developed. ***Colouration in fresh***. Colouration shown in Fig. 3. Head and body yellowish brown, with irregular dark-brown spots. Branchiostegal membrane bright yellow. Base of lateral scales dark, forming a reticular pattern on body. Belly pale yellow. Males darker than females. Wide blackish stripe from eye to lower lip, faint in females. First dorsal fin transparent; spinous rays dark brown; distal area yellow; males with 3–4 black marks on anterior membranes, 1–2 longitudinal rows of brown spots; females with 4–5 longitudinal rows of brown spots. Second dorsal fin transparent, with 9–11 longitudinal rows of inverted V-shaped or dash-like brown markings; membrane edge yellow. Anal fin greyish yellow, with a narrow white margin. Caudal fin yellow, with 6–7 vertical rows of brown spots. Pectoral fin membrane light yellow. Pelvic fin yellow.

##### Etymology.

The species is named after its type locality, the Mengyang Town.

##### Vernacular name.

濛阳吻虾虎鱼 (méng yáng wěn xiā hǔ yú).

##### Distribution and habitat.

The new species is currently reported from the Tuojiang River and the Minjiang River, two tributaries of the Changjiang River (Fig. 6). It occurs in pebble-bottomed sections of cold, flowing rivers; in sympatry with *Acheilognathus mengyangensis* Chen, Gong & Guo, 2021, *Gobiocypris rarus* Ye & Fu, 1983, *Yunnanilus jiuchiensis* Du, Hou, Chen & Yang, 2018, *Opsariichthys chengtui* Kimura, 1934, and *Belligobio pengxianensis* Lo, Yao & Chen, 1977. In the Minjiang River basin, the new species occasionally occurs in sympatry with *R. szechuanensis*, but no *R. szechuanensis* has been recorded from the Tuojiang River basin.

## Discussion

Sympatric freshwater gobies usually exhibit microhabitat differentiation (Li 2011). In the present study, *R. mengyangensis* sp. nov. and *R. szechuanensis* occur sympatrically in the Minjiang River basin, representing a sympatric sister-species pair. A similar sympatric sister pair has been reported in *Liobagrus pseudostyani* and *L. brevispina* from the Minjiang and Tuojiang River basins (Chen et al. 2022b). Interestingly, in *R. mengyangensis* sp. nov., populations from the Qingyijiang River basin (a tributary of the Min River) possess 16–17 pectoral-fin rays, compared with 19–21 in populations from other river basins (all specimens from Chengdu; Suppl. material 2: table SS2). In addition, *R. mengyangensis* sp. nov. exhibits slight genetic differentiation in *Cyt b* gene between populations from the ​​Qingyijiang River basin and other river basins (Fig. 1, genetic distance ≈ 0.02). Given that *R. mengyangensis* sp. nov. is only found in one locality of the Minjiang River basin near Chengdu, which is outside its main range in the Qingyijiang and Tuojiang basins, its presence in this area is likely due to a recent hydrological connection between the Minjiang and Tuojiang Rivers. These results suggest that the populations of *R. mengyangensis* sp. nov. in the Qingyijiang River basin and those from other river basins may have undergone distinct periods of independent evolutionary divergence.

**Figure 7.**
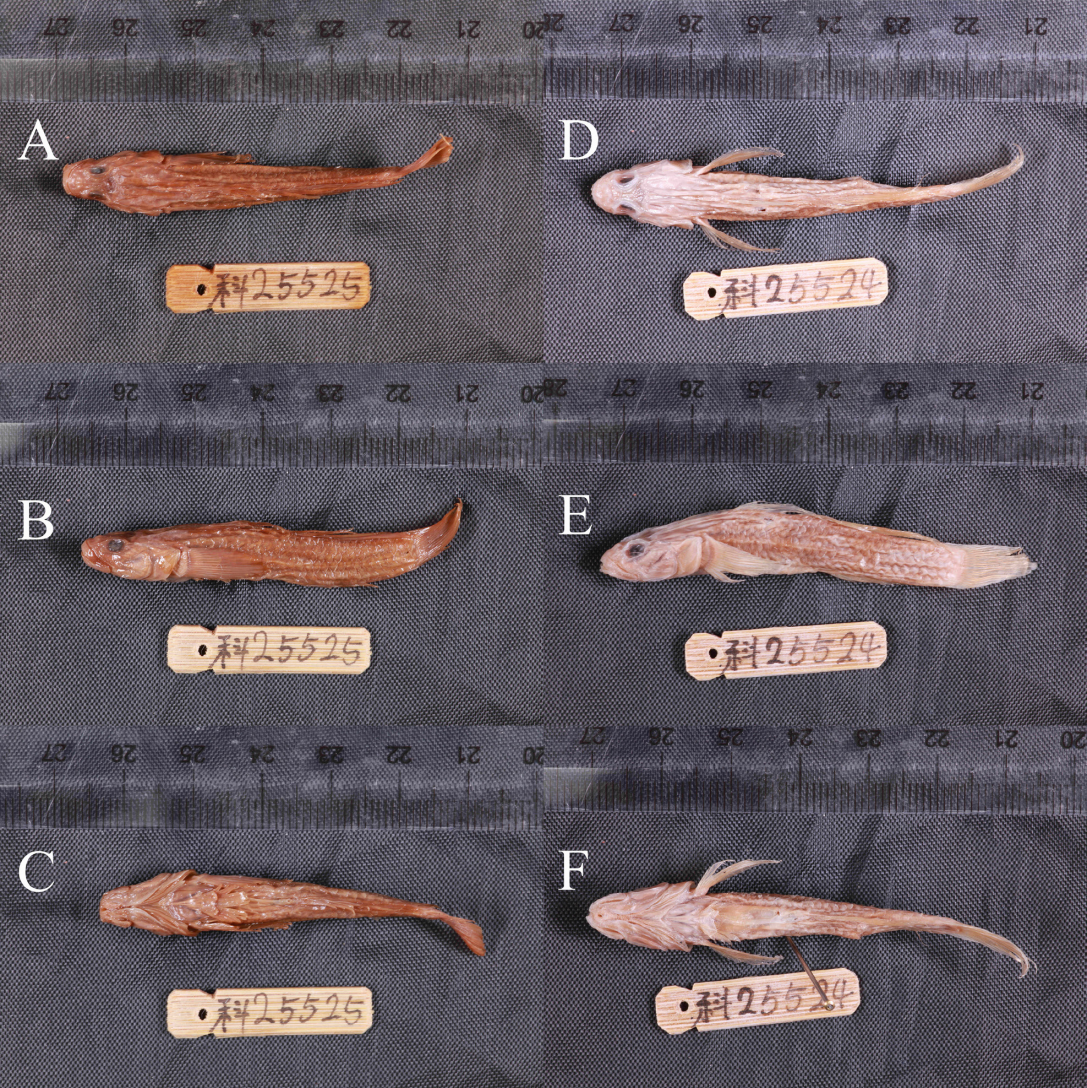
*
Rhinogobius szechuanensis*, type specimens. **A–C**. Holotype, ASIZB 25525; **D–F**. Paratype, ASIZB 25524.

The new species was considered to be a yellow type of *R. szechuanensis* (samples from Yibin) (Guo et al. 2021). Some characters of the type specimens of *R. szechuanensis* were obscured by dehydration during preservation. Both specimens (from Chengdu, Fig. 7) exhibit fewer pectoral-fin rays (17), which distinguish them from *R. mengyangensis* sp. nov. from Chengdu (19–21 rays) (Table 3). In living animals, *R. mengyangensis* sp. nov. exhibits second dorsal fin with 9–11 longitudinal rows of inverted V-shaped or dash-like brown markings (vs with blue spots, some coalescing into vertical bars), incomplete brownish edges on flank scales below second dorsal fin (vs with complete edges), a bright-yellow branchiostegal membrane (vs white or blue), which distinguish it from *R. szechuanensis* (Fig. 5). Predorsal length in *R. mengyangensis* (37.92–43.18%) tends to be slightly larger than in *R. szechuanensis* (35.57–37.39%), although the ranges are close, and this character alone is not considered diagnostic.

**Table 3.** Comparison of morphological and meristic measurements between *Rhinogobius mengyangensis* sp. nov., *R. szechuanensis*, and *R. chengtuensis*.

**Species**	*** R. mengyangensis* sp. nov**.		*** R. szechuanensis***			*** R. chengtuensis* = *R. szechuanensis***
Number of samples	Holotype	*n* = 15; min–max (mean)	Holotype	Paratype	*n* = 7 (from present study); min–max (mean)	Syntype; *n* = 3; min–max (mean)
Morphometrics characters						
Standard length (mm)	53.17	37.68–59.32 (46.66)	56.80	64.20	32.9–49.4 (40.09)	39.83–49.76 (44.23)
Head length (mm)	16.87	10.5–18.58 (14.3)	14.40	14.50	9.3–13.6 (11.34)	10.75–13.93 (12.04)
Percent standard length (%)						
Head length	31.73	26.18–33.83 (30.55)	27.91	25.48	26.46–31.41 (28.26)	26.56–27.99 (27.18)
Predorsal length	40.42	37.92–43.18 (40.06)	37.21	37.43	35.57–37.39 (36.29)	37.16–37.39 (37.28)
Snout to second dorsal fin origin	58.89	55.82–63.65 (59.12)	50.97	50.97	52.4–57.97 (56.66)	54.94–58.83 (57.08)
Snout to anus	63.55	55.82–66.16 (61.53)	59.89	58.17	53.89–59.35 (55.60)	54.34–57.69 (55.96)
Prepelvic length	30.3	25.69–34.41 (30.86)	26.94	28.30	27.78–30.02 (29.07)	28.25–31.51 (29.84)
Caudal peduncle length	19.86	17.97–24.88 (21.64)	16.86	18.10	20.91–24.07 (22.48)	19.47–22.02 (21.02)
Caudal peduncle depth	13.11	10.92–13.11 (12.2)	10.74	9.81	10.32–13.63 (11.78)	11.78–12.18 (12.00)
First dorsal fin base	18.79	12.99–18.79 (16.03)	10.07	12.30	11.68–17.32 (14.91)	12.2–14.41 (13.46)
Second dorsal fin base	18.83	17.58–25.87 (20.52)			16.4–22.75 (19.23)	18.15–19.47 (18.62)
Anal fin base	14.9	10.28–19.06 (14.67)			11.11–16.83 (13.65)	16.22–19.98 (18.20)
Caudal fin length	21.12	17.74–24.85 (21.82)			20.24–25.23 (22.54)	23.24–26.43 (24.57)
Pectoral fin length	22.23	20.38–23.53 (22.06)	25.78	23.90	20.85–24.25 (22.56)	20.86–24.03 (22.18)
Pelvic fin length	14.99	11–22.35 (15.23)			13.49–17.63 (15.66)	12.02–13.57 (12.90)
Body depth of pelvic fin origin	16.02	13.34–21.81 (16.84)	12.85	14.17	16.4–19.46 (18.10)	12.13–13.58 (12.77)
Body depth of anal fin origin	15.82	13.34–19.51 (16.12)	12.50	12.15	14.55–17.37 (15.68)	14.71–15.79 (15.14)
Pelvic fin origin to anus	32.2	25.87–36.89 (29.58)	26.23	26.64	22.75–27.71 (25.56)	26.47–28.97 (27.53)
Head depth	18.11	12.91–18.71 (15.87)	12.32	13.55	13.49–17.37 (15.98)	12.08–12.6 (12.28)
Percent head length (%)						
Snout length	25.31	19.01–26.67 (23.35)	25.00	36.55	19.15–25.93 (22.58)	20.47–27.78 (24.20)
Eye diameter	19.62	15.33–26.62 (19.94)	15.97	21.38	17.78–23.66 (20.15)	21.32–25.15 (23.55)
Cheek depth	26.32	14.93–31.88 (21.56)	28.47	21.38	12–22.96 (18.04)	28.3–30.94 (29.45)
Postorbital length	60.58	49.64–60.95 (57.09)	52.78	55.86	48.94–55.56 (51.68)	51.79–54.6 (53.39)
Lower jaw length	38.65	22.78–39.78 (30.03)	30.56	35.86	22–28.68 (24.66)	24.72–28.79 (26.45)
Interorbital width	20.98	8.25–22.48 (16.51)	13.89	15.17	9.68–17.65 (14.10)	12.23–18.59 (14.43)
Head width in maximum	78.3	57.93–78.91 (68.09)	67.36	70.34	63.24–81.91 (69.74)	58.65–66.2 (61.96)
Meristic counts						
First dorsal fins	VI	VI (15)			V (2), VI (5)	VI (3)
Second dorsal fins	I, 9	I, 8 (2), I, 9 (11), I, 10 (2)			I, 8 (1), I, 9 (6)	I, 8 (2), I, 9 (1)
Anal fins	I, 8	I, 7 (10), I, 8 (3), I, 9 (2)			I, 7 (2), I, 8 (5)	I, 7 (2), I, 8 (1)
Pectoral fins	19	16 (5), 17 (2), 19, (1)20 (5), 21 (2)	17	17	17 (6), 18 (1)	17 (3)
Longitudinal scales	31	30 (1), 31 (2), 32 (4), 33 (4), 34 (2), 35 (2)	33	32	26 (1), 29 (2), 31 (3), 32 (1)	32 (1), 33 (1), 34 (1)
Transverse scales	10	9 (11), 10 (4)	8	8	8 (1), 9 (2), 10 (4)	10 (2), 11(1)
Predorsal scales	6	5 (2), 6 (2), 8 (2)			5 (1), 7 (1), 8 (1)	

**Figure 8.**
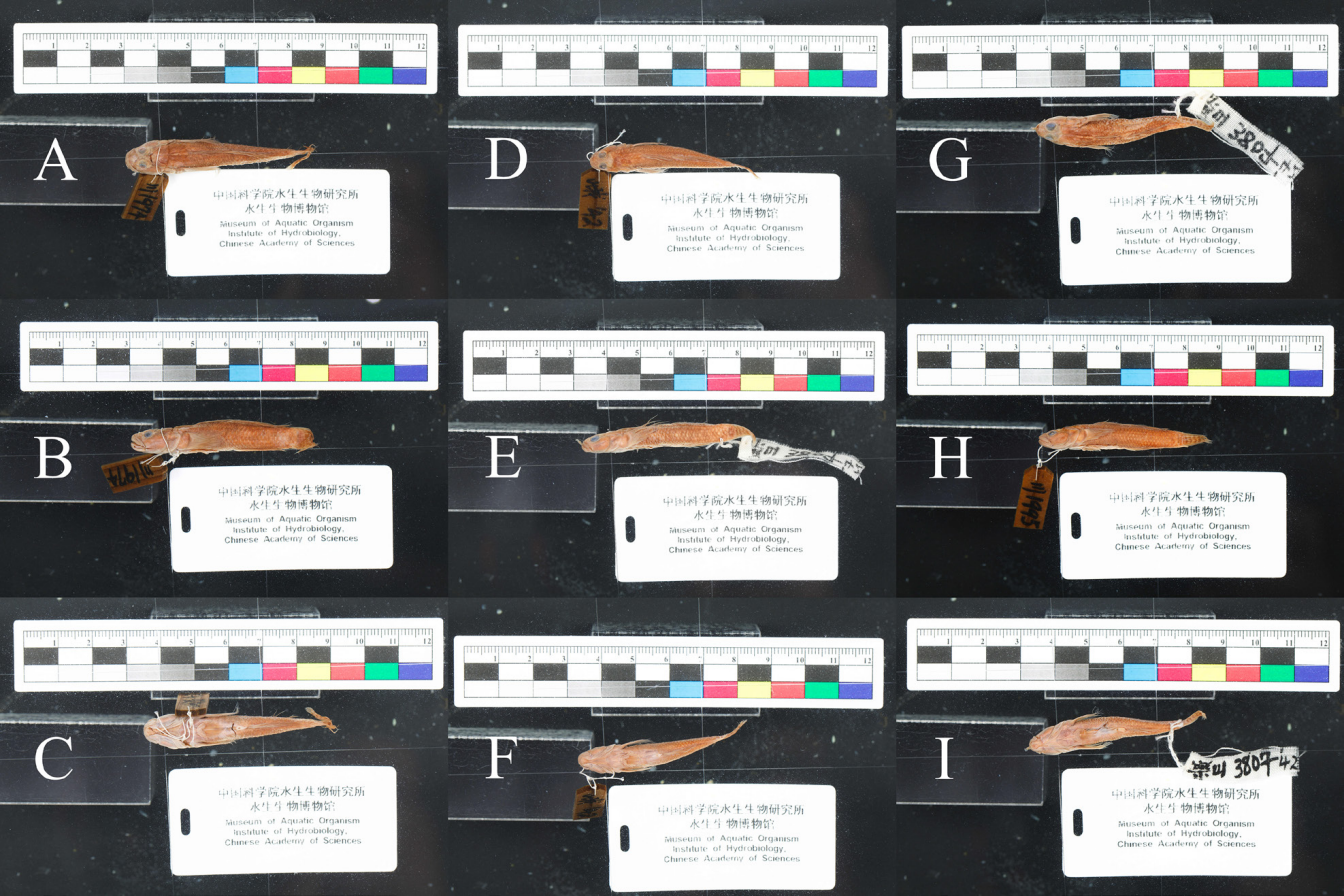
*
Rhinogobius chengtuensis*, type specimens. **A–C**. No. 1974, male; **D–F**. No. 1995, female; **G–I**. No. 3807, male.

*Rhinogobius chengtuensis* Chang, 1944 was treated as a junior synonym of *R. szechuanensis* (Wu and Zhong 2008). Its type specimens (from Emeishan) exhibit complete brownish edges on flank scales below second dorsal fin, which are consistent with *R. szechuanensis* but differ to *R. mengyangensis* sp. nov. (Fig. 8). The type specimen of *R. chengtuensis* from Chengdu (No. 1609) is missing; however, according to the original description, *R. chengtuensis* possesses 16–18 pectoral-fin rays (Chang 1944), which is inconsistent with *R. mengyangensis* sp. nov. from Chengdu (19–21 rays). Given that *R. chengtuensis* was recorded from Chengdu and Emeishan, phylogenetic analysis of the *Cyt b* gene in this study supports the assignment of specimens from the Emeishan region to *R. szechuanensis*. Furthermore, our phylogenetic results indicate that no third species, sister to either *R. szechuanensis* or *R. mengyangensis* sp. nov., occurs in the Chengdu region. Accordingly, we agree that *R. chengtuensis* is a junior synonym of *R. szechuanensis* and does not represent a valid species.

Mengyang Town, the type locality of *R. mengyangensis* sp. nov., is also the type locality of two other recently described freshwater fish species, *Acheilognathus mengyangensis* Chen, Gong & Guo, 2021 and *Yunnanilus jiuchiensis* Du, Hou, Chen & Yang, 2018. Moreover, in recent years, several new freshwater fish species, including *Rhodeus cyanorostris* Li, Liao & Arai, 2020 and *Liobagrus chengduensis* Chen, Guo, Wu & Wen, 2022, as well as a new freshwater mussel species, *Pseudocuneopsis sichuanensis* Wu, Liu & Ouyang, 2022, have been discovered in the Chengdu region, indicating that freshwater biodiversity in this area has been underestimated. At present, riverine ecosystems in the Chengdu region are highly susceptible to degradation, underscoring the urgent need for effective conservation strategies to protect these waterways.

## Supplementary Material

XML Treatment for Rhinogobius mengyangensis

## Additional information

### Artificial Intelligence (AI) use

The authors accept full responsibility for the content of the manuscript, including the disclosure of any use of AI.

No AI tools were used in the preparation of this manuscript.

### Author ORCIDs

X. Liu https://orcid.org/0009-0003-1494-9540

Z-G. Chen https://orcid.org/0000-0003-2689-3321

J-H. Huang https://orcid.org/0009-0004-0671-1003

### Data availability

All of the data that support the findings of this study are available in the main text or Supplementary Information.

## Supplementary materials

Supplementary material 1 Supplementary material 1: table SS1 Data type xlsx Explanation note Raw morphometric measurements of *R. szechuanensis* and *R. chengtuensis* type specimens. https://binary.pensoft.net/file/1661310This dataset is made available under the Open Database License (http://opendatacommons.org/licenses/odbl/1.0/). The Open Database License (ODbL) is a license agreement intended to allow users to freely share, modify, and use this Dataset while maintaining this same freedom for others, provided that the original source and author(s) are credited. Xin Liu, Zhong-Guang Chen, Yi-Fan Shu, Jun-Hao Huang, Kai Liu, Yi Yu

Supplementary material 2 Supplementary material 1: table SS2 Data type xlsx Explanation note Comparison of morphological and meristic measurements in *R. Mengyangensis* between Qingyijiang River basin and other river basins. https://binary.pensoft.net/file/1661311This dataset is made available under the Open Database License (http://opendatacommons.org/licenses/odbl/1.0/). The Open Database License (ODbL) is a license agreement intended to allow users to freely share, modify, and use this Dataset while maintaining this same freedom for others, provided that the original source and author(s) are credited. Xin Liu, Zhong-Guang Chen, Yi-Fan Shu, Jun-Hao Huang, Kai Liu, Yi Yu
